# Promoting Later Life Meaningful Engagement: A case study of Tech-Assisted social enterprise in Hong Kong

**DOI:** 10.1093/geroni/igaf122.2477

**Published:** 2025-12-31

**Authors:** Shiyu Lu

**Affiliations:** The University of Hong Kong, Hong Kong, China (People’s Republic)

## Abstract

**Background and Objectives:**

Meaningful engagement, defined as participation in enjoyable activities tailored to individual interests and abilities, is a critical factor in promoting a healthy and fulfilling later life for older adults. However, there is limited understanding of how to effectively foster such engagement in this population. Research Design and

**Methods:**

This study explores the innovative features of Happy Friend, a social enterprise project that engages older adults in diverse social and productive activities. Data were collected through one in-depth interview with the founder, two focus group interviews with staff, and six interviews with older adult participants. Thematic analysis was used to analyze the interview content.

**Results:**

The study identified a three-tiered stepped-up model for promoting meaningful engagement: 1) Joyful Community-Based Group Activities to strengthen social connections; 2) Role Transition and Empowerment, encouraging older adults to take on roles such as mentors to foster a sense of purpose; and 3) Self-Actualization and Societal Contribution, leveraging the skills of older adults who excel in leisure activities to combat ageism through collaborative partnerships. Additionally, key enablers of this model were identified, including co-production mechanisms, a low-cost technology-assisted platform to enhance autonomy for both providers and recipients, and a time-credit-based system to recognize talents and encourage sustained engagement.

**Discussion and Implications:**

These findings highlight the potential of a structured, strengths-based approach to fostering meaningful engagement in later life, offering practical insights for policymakers, practitioners, and communities aiming to enhance the well-being of older adults.

